# Bilateral Papillary Traction due to Persistent Hyaloid Vessel Remnants in Aymé–Gripp Syndrome

**DOI:** 10.1155/crop/2557037

**Published:** 2026-08-10

**Authors:** Carolina Carvalho Soares Valentim, Mohamed M. Khodeiry, Maja Kostic

**Affiliations:** ^1^ Department of Ophthalmology and Visual Sciences, University of Louisville, Louisville, Kentucky, USA, louisville.edu; ^2^ Bascom Palmer Eye Institute, University of Miami Miller School of Medicine, Miami, Florida, USA, miami.edu; ^3^ Bascom Palmer Eye Institute, Ophthalmology Department, University of Miami Miller School of Medicine, Miami, Florida, USA, miami.edu

## Abstract

**Introduction:**

Although papillary traction often arises from anomalous vitreous detachment, it can be related to traction from persistent remnants of the fetal hyaloid vasculature.

**Case Presentation:**

A 17‐year‐old girl with Aymé–Gripp syndrome was incidentally found to have bilateral optic nerve elevation in the setting of bilateral persistent hyaloid vessel remnants causing papillary traction. Brain imaging was unremarkable.

**Conclusion:**

This case highlights the rare occurrence of bilateral papillary traction in a young patient with a genetic syndrome and underscores the importance of considering papillary traction in the differential diagnosis of optic nerve elevation.

## 1. Introduction

Papillary traction is characterized by tissue adhesion to the optic nerve, which exerts traction on it, often presenting as pseudopapilledema [1]. This phenomenon is often associated with age‐related incomplete posterior vitreous detachment (PVD) or fibrocellular tissue contraction secondary to diseases such as diabetes mellitus. Less commonly, nonregressed remnants of the fetal hyaloid vasculature, known as Bergmeister papilla, may exert traction on the optic nerve through persistent adherence to it [2]. Clinical manifestations range from asymptomatic to severe, with the potential for permanent vision loss.

## 2. Case Report

A 17‐year‐old female with a history of Aymé–Gripp syndrome was referred for a neuro‐ophthalmology evaluation after an incidental finding of bilateral optic nerve edema. Her medical history included Aymé–Gripp syndrome, attention‐deficit/hyperactivity disorder (treated with amphetamine extended release 9.4 mg), anxiety (fluoxetine 40 mg), ovarian cyst (norgestrel‐ethinyl estradiol 0.3–30 mg‐mcg), eosinophilic esophagitis (budesonide 1 mg/2 mL), and hidradenitis suppurativa (doxycycline 200 mg). Her ophthalmologic history included high myopia, bilateral infantile cataract with prior bilateral cataract extraction with posterior chamber intraocular lens (PCIOL) implantation 12 years ago, bilateral ocular hypertension treated with a Baerveldt glaucoma implant (BGI) in the right eye 4 years ago, and longstanding diplopia likely due to restrictive right hypotropia secondary to the glaucoma drainage device. Intraocular pressure was controlled with dorzolamide–timolol combination eyedrops in both eyes.

**Figure 1:**
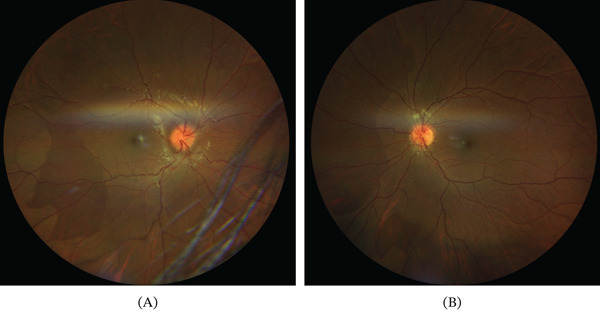
Fundus photographs demonstrating bilateral Bergmeister papillae and optic nerve elevation, more pronounced in (A) the right eye compared with (B) the left eye.

The patient reported right eye throbbing pain associated with nonpositional, pressure‐like headache for a few days but denied tinnitus, transient visual obscurations, or recent weight change. Initial ophthalmological exam demonstrated best corrected visual acuity of 20/25 in both eyes with full color plate vision, equal and round pupils with brisk reaction, no relative afferent pupillary defect, full confrontational visual fields, right hypotropia of four prism diopters, and intraocular pressures of 12 in the right eye and 19 mmHg in the left eye. Anterior segment showed a well‐positioned right BGI, right peripheral anterior synechiae, and well‐centered bilateral PCIOL. Posterior segment exam revealed bilateral Bergmeister papillae and bilateral optic nerve elevation, more pronounced in the right eye (Figure 1).

**Figure 2:**
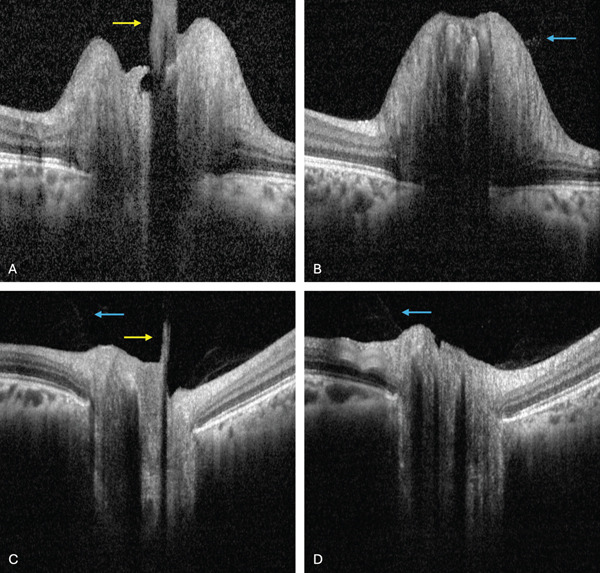
Optic coherence tomography (Spectralis SD‐OCT, Heidelberg Engineering) raster scans of the (A‐B) right and (C‐D) left optic nerves demonstrating Bergmeister papilla and vitreopapillary traction.

**Figure 3:**
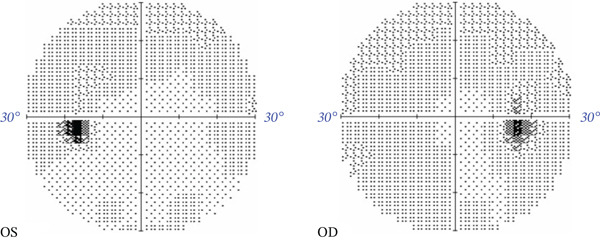
Automated Humphrey perimetry showing full visual field in the right eye and few superior, nonspecific defects in the left eye.

The patient was referred to the emergency department for urgent evaluation. MRI of the brain and orbits (with and without contrast) and brain MRV were normal. Opening pressure was 21 cm H_2_O with normal CSF analysis. Follow‐up neuro‐ophthalmology visit included repeated OCT raster scans over the optic nerves, which showed bilateral vitreopapillary traction (Figure 2); and automated Humphrey perimetry that was unreliable but overall full in the right eye, and demonstrated few superior, nonspecific defects in the left eye (Figure 3). Observation was recommended given the patient′s lack of visual symptoms.

## 3. Discussion

This case presents an unusual occurrence of bilateral papillary traction in a young patient with Aymé–Gripp syndrome, a rare genetic disorder associated with a heterozygous pathogenic variant of the *MAF* gene (OMIM 177075), which encodes for a transcription factor involved in morphogenesis and neurodevelopment [3]. Aymé–Gripp syndrome classically presents with a combination of bilateral early cataracts, sensorineural hearing loss, distinct facial features including brachycephaly and midfacial hypoplasia, and developmental delay [4]. Retinal and optic nerve abnormalities have also been documented [5].

The pathophysiology of papillary traction in this patient is likely related to the persistent hyaloid vessel remnants causing traction at the optic nerves. The association between Bergmeister papilla, optic nerve elevation, and retinal traction leading to vitreomacular traction syndrome has been reported [2, 6]. Additionally, a traction‐related phenomenon at the optic nerve head has been suggested as the mechanism underlying the strong association between Bergmeister papilla and increased prelaminar schisis severity in both normal and glaucomatous eyes [7].

The finding of bilateral optic nerve elevation in this patient led to urgent neurological evaluation, which ruled out other potential causes. This case highlights the importance of considering papillary traction in young patients presenting with optic nerve elevation, particularly when they have a history of ocular or systemic abnormalities.

## Funding

No funding was received for this manuscript.

## Disclosure

All authors have read and approved the final version of the manuscript. Maja Kostic had full access to all of the data in this study and takes complete responsibility for the integrity of the data and the accuracy of the data analysis. This study was conducted at the Department of Ophthalmology and Visual Sciences, University of Louisville.

## Ethics Statement

The authors have nothing to report.

## Consent

Informed written consent was obtained from the patient′s parent.

## Conflicts of Interest

The authors declare no conflicts of interest.

## General Statement

*Transparency Statement*. The manuscript guarantor, Maja Kostic, affirms that this manuscript is an honest, accurate, and transparent account of the study being reported; that no important aspects of the study have been omitted; and that any discrepancies from the study as planned (and, if relevant, registered) have been explained.

## Data Availability

The data that support the findings of this study are available from the corresponding author upon reasonable request.
